# Existence of late-effects instruments for cancer survivors: A systematic review

**DOI:** 10.1371/journal.pone.0229222

**Published:** 2020-02-24

**Authors:** Hillary Klonoff-Cohen, Mounika Polavarapu

**Affiliations:** 1 Department of Kinesiology and Community Health, University of Illinois Urbana-Champaign, Champaign, Illinois, United States of America; 2 School of Population Health, University of Toledo, Toledo, Ohio, United States of America; Karolinska Institutet, SWEDEN

## Abstract

**Introduction:**

The number of cancer survivors is projected to increase to 22.1 million by 2030. Late effects incorporate the full domains of cancer survivorship (e.g., physiologic, psychosocial, economic). They are numerous, complex, and potentially alter the life trajectories of cancer survivors. Currently, research is missing on the impact of late effects (e.g., cardiomyopathy, fertility, lymphedema, anxiety) on cancer survivors.

**Objective:**

The goal of this study is to present a systematic review of existing instruments for identifying, diagnosing, and managing late effects within cancer survivors.

**Methods:**

Using PRISMA guidelines, a systematic search was conducted using the electronic databases of PubMed and Web of Science to identify relevant papers. Articles considered eligible for this review met the following criteria: 1) written in English, 2) published until September 30, 2019, and 3) containing instruments with questions on late effects. Hypothesis, study design, study sample, questionnaire domains, details of late effects, results, conclusions, and advantages/disadvantages of each article were assessed using a modified version of the NHLBI quality assessment tool.

**Results:**

An exhaustive literature review revealed 576 publications in PubMed, 628 in Web of Science, and 260 from additional sources. After removing duplicates, articles without late-effects questionnaires, and publications using identical questionnaires, 11 studies fulfilled the eligibility criteria. Study quality assessment was measured on a scale of 0–6 (0 = poor quality; 6 = highest quality). Only one study was rated with a score of 5 (Rocque).

**Conclusions:**

Taken in totality, none of the studies adequately addressed the prevalence, etiology, characteristics, management, and prevention of late effects. There is currently no comprehensive questionnaire that captures all of the relevant details of late effects across the cancer survivorship continuum nor that tracks the interrelatedness of multiple late effects. Hence, it is difficult to identify, diagnose, manage, and ultimately prevent late effects.

## Introduction

There are 16.9 million cancer survivors in the United States, representing 5% of the population [1]. The number of cancer survivors is projected to increase to 22.1 million, by 2030 [1]. Nearly two-thirds of these individuals are 65 years or older [1]. As the population of cancer survivors in the US grows, it becomes essential to optimize health care delivery and long-term outcomes among survivors [2]. People with a history of cancer have unique medical and psychosocial needs that require proactive assessment and management by their oncologists and primary care providers [1]. Cancer survivors deal with both long-term effects and late effects.

Long-term effects refer to any side effects or complications of treatment for which a cancer patient must compensate; they begin during treatment and continue beyond the end of treatment [3] (heart, lung, kidney, or gastrointestinal tract problems; pain, numbness, tingling, loss of feeling, or heat or cold sensitivity in the hands or feet; fatigue; metabolic syndrome; bone loss; hearing loss; cataracts; dry eyes or dry mouth; and financial toxicity) [4,5]. Late effects refer specifically to unrecognized toxicities that are absent or sub-clinical at the end of therapy and manifest later [3]. These effects appear months to years after the completion of treatment [5] and their risk increases over time [6,7]. Currently, three out of five survivors develop late effects [8]. While most late effects are not life-threatening, they may cause serious problems that affect health and quality of life (physical, psychological, social, and spiritual wellbeing) [3,7]. Late effects are rarely examined in isolation; rather they incorporate the full domains of cancer survivorship (e.g., physiologic, psychosocial, economic) [6]. Adverse late effects can be numerous and complex and potentially alter the life trajectories of cancer survivors [9]. Hence, survivors can struggle daily with: a) the physical sequelae of late effects (e.g., cardiomyopathy, central nervous system problems [thinking, learning, memory, fatigue], sexual health and fertility, and lymphedema) as well as risks of cancer recurrences, b) psychosocial sequelae of late effects such as anxiety, depression, relationship complications, body image disturbances, and poor self-esteem, and c) financial consequences of late effects including unemployment, medical insurance, and finances [6, 10–12]. Research is urgently needed into the impacts of late effects on the physical and psychological health and quality of life of cancer survivors.

### Objectives

The goal of this study is to present a systematic review of existing instruments for identifying, diagnosing, and managing late effects within cancer survivors.

## Methods

This review was conducted following the guidelines outlined in the Preferred Reporting Items for Systematic Reviews and Meta-Analyses: The (PRISMA) Statement [13] (S1 Fig).

### Search strategy

A systematic search was performed using the electronic database PubMed to discover all relevant articles. The search included the keywords: late effects, cancer survivors, instrument, questionnaire, and survey. A second search was completed using the same keywords in the Web of Science to ensure attaining all relevant articles. Additionally, articles located in the bibliography of the relevant papers were reviewed for significance. All reported studies that used the key words “late effects” as well as those that discussed late effects in their introduction and abstract were included.

### Eligibility criteria

Eligible articles met the following criteria: i) written in English, ii) original articles published until September 30, 2019, and iii) containing instruments with questions on late effects.

### Study selection

Titles, abstracts, and keywords were screened to confirm reliability with the eligibility criteria. All articles were examined to identify presence of late effects questions within the study questionnaires. Only those relevant articles were included in this review.

### Data collection process and data items

The selection process of studies was performed independently by two reviewers (HKC and MP). The author, hypothesis, study design, study sample, sample size, questionnaire, domains of questionnaire, details of late effects, results, conclusions, and advantages and disadvantages of each article are presented in this systematic review.

### Study quality assessment criteria

A thorough independent assessment of the methodological quality was also performed by two authors (HK and MP). The NHLBI Quality Assessment of Systematic Reviews and Meta-Analyses tool contains 8 questions with no total score. A modified version of this tool was used in this study which consisted of the following questions: i) Was the study question or objective clearly stated? Ii) Were the subjects comparable within each study? Iii) Were the outcome measures clearly defined, valid, reliable, and implemented consistently across all study participants? Iv) Were details regarding the exposure provided? V) Was the questionnaire relevant to late effects? Vi) Was the length of follow-up adequate? The original NHLBI Quality Assessment of Systematic Reviews and Meta-Analyses consists 8 questions with no total scores, rated as yes or no by two independent reviewers. The best evidence synthesis was performed rating qualities of the studies using dichotomous criteria (i.e., 0 = no, and 1 = yes).

## Results

**Fig 1:**
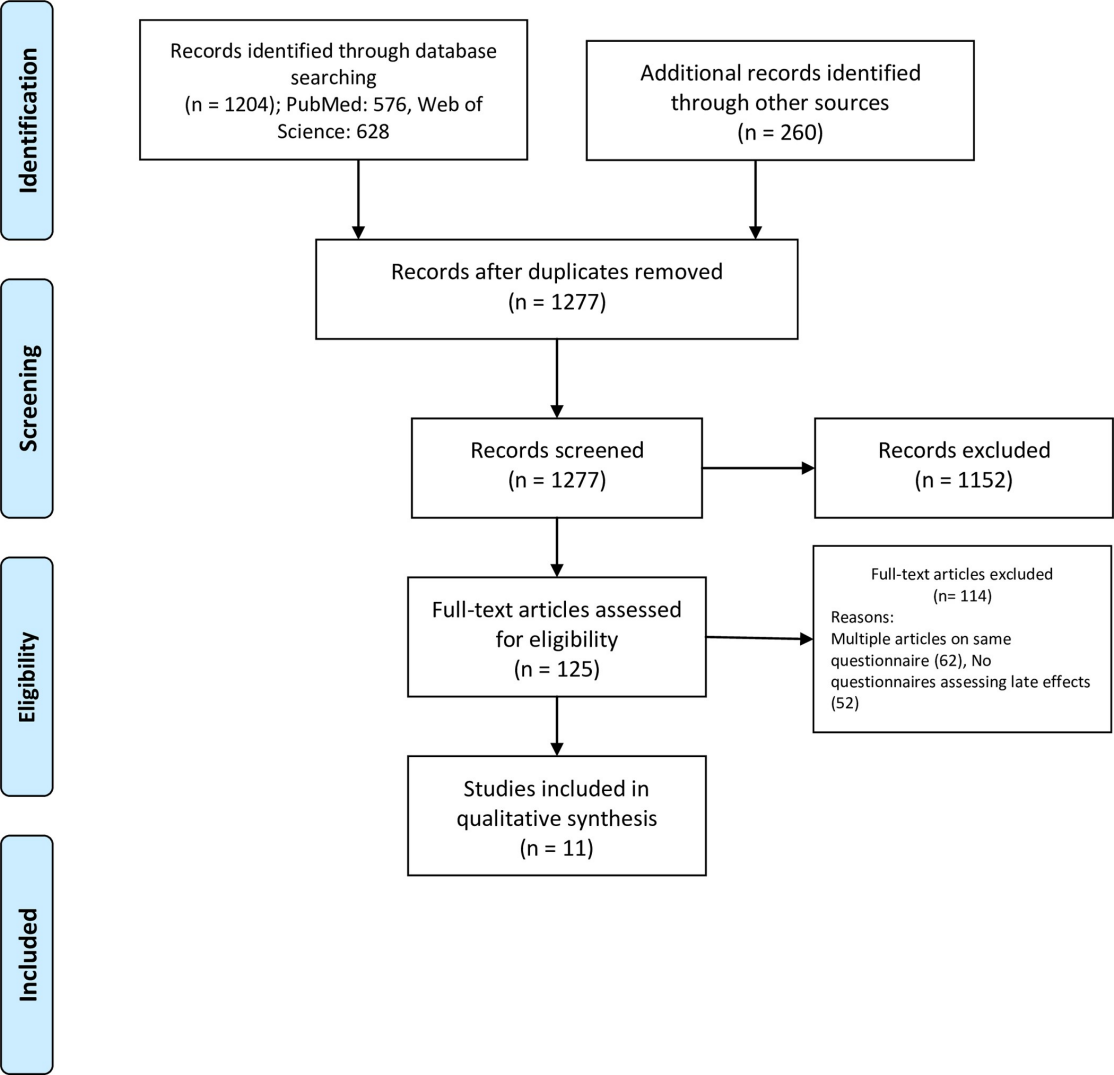
PRISMA flowchart for demonstrating the process of identification, assessment, and inclusion of studies in the systematic review.

An exhaustive review of the literature revealed a total of 576 publications in PubMed and 628 in Web of Science. The reference list of related review articles resulted in an additional 260 relevant records. One-hundred eighty seven duplicates were deleted using EndNote bibliographic software. Upon elimination of duplicates, a thorough screening of titles, abstracts, and keywords was implemented yielding 1277 articles (Fig 1). A total of 125 full text articles were then reviewed against the eligibility criteria. One hundred and fourteen articles were eliminated because they: i) did not contain a questionnaire, ii) were one of multiple publications using the same questionnaire and data already captured, and iii) did not study late effects among cancer survivors. In the end, eleven studies fulfilled the requirements of the eligibility criteria [14–24]. All the studies included in this systematic review are summarized and presented in Table 1.

**Table 1:** Characteristics of studies included in the systematic review.

Author	Hypothesis	Study Design	Sample Characteristics	Sample Size	Questionnaire	Domains	Late Effects Presented	Results	Conclusions	Advantages and Disadvantages
Cancer Experie-nce Registry 2017 [14]	1) To understand the psychosocial experiences and needs of people who have been impacted by cancer; 2) To inform the research community, healthcare providers, patient advocates and policy makers around gaps in care and the challenges of people affected by cancer.	Cross-sectional and longitude-nal	Over 12,000 participants- survivors, patients across the cancer experience, and caregivers. Target study sample is 15,000. Recruiting until 2035 Over 45 cancer types are included in the registry	Greater than 12,000 survivors, patients, and caregivers	Unnamed questionnaire containing demographics and background, cancer-related distress, quality of life, treatment decision-making and planning, side effects and symptom management, financial problems, and work-related experience	Social, emotional, physical, financial and decision-making experiences of those who have been diagnosed with cancer and their caregivers	Side effects were presented but with no late effects	The 2017 results revealed quality of life (39% rated their QOL as very good or excellent), treatment decision-making (24% did not feel prepared to discuss treatment with their doctor), side effects and management (1 in 5 reported the health care team did not explain short-term side effects), and financial problems (73% did not talk about costs with the care team).	Although short term side effects and quality of life were assessed, late-effects were omitted.	Large sample size Large amount of cancer diagnoses Inclusion of caregivers and stakeholders Long follow-up period The major disadvantage is that this questionnaire did not address late-effects at all.
The Childhood Cancer Survivor Study (CCSS) [15]	To characterize the experience of participants regarding late-effects and other delayed effects of treatment.	Retrospective cohort	Individuals who survived five or more years after diagnosis of cancer, leukemia, tumor, or similar illness diagnosed during childhood or adolescence.	The CCSS is a retrospective cohort of 35,923 childhood cancer survivors. Baseline data was collected on 14,054 survivors and ompreh.-imately 5,000 siblings	CCSS questionnaire	i) Genetic conditions, ii) conditions at birth, iii) medications, iv) radiation treatment for a cancer recurrence, v) pregnancy and offspring, vi) family history of cancer, vii) medical conditions by system (respiratory, cardiovascular, nervous system), viii) fatigue/sleeping, ix) health insurance, xiii) diagnosed with another cancer, leukemia, tumor, or recurrence (relapse).	No information on content of late effects other than inquiring about cardiac, respiratory, nervous, and hepatic systems.	14,054 subjects completed a 24-page baseline questionnaire. The survivors diagnosis included leukemia (33%), lymphoma (21%), neuroblastoma (7%), central nervous system tumors (13%), bone tumor (8%), kidney (9%), and soft tissue sarcoma (9%). A total of 78% received radiation and 73% receive chemotherapy.	The CCSS serves as a database for addressing long term effects such as risk of second malignancies, endocrine and reproductive outcome, cardiopulmonary complications, and psychosocial implications, among this unique and ever-growing population.	The CCSS represents the largest cohort of child- hood and adolescent cancer survivors in North America. Self-reported diagnoses, small number of non-Caucasian survivors. There were several organs omitted (e.g., gastrointestinal, genitourinary, central nervous system, musculoskeletal, thyroid, lymphatic, immune) from the CCSS questionnaire on late effects, the questions on late effects were superficial (i.e., present or absent), and only included childhood cancer survivors.
Curcio, 2012 [16]	To implement and evaluate a survivorship protocol for cancer survivors to improve their knowledge and decrease their anxiety Secondary outcomes were to evaluate the satisfaction of survivors, staff, and PCPs with the program	Pre-Post test 1 month later	Thirty survivors from one community cancer center who completed cancer treatment within the past 2 years, and were >18 years.	A convenience sample of 30 cancer survivors	A baseline anxiety score using GAD-7 scale and survivor knowledge using a knowledge questionnaire. One-month later anxiety level and knowledge were re-assessed.	Survivorship knowledge including diagnosis, treatment, signs of recurrence, and late side effects	Patients express anxiety about managing late side effects. They report feeling unprepared for transition from being a patient to a survivor.	One month after the survivorship protocol was delivered, knowledge about diagnosis, treatments, recommended follow-up, signs of recurrence, and late side effects increased. A total of 40% (4/10) participants were able to name at least one late side effect at baseline which increased to 69% (18/26) at one-month follow-up. Anxiety scores were lower one month after the intervention, and satisfaction with the protocol was high.	Survivors found the intervention to be very helpful and were satisfied with it.	The survivorship protocol increased knowledge about diagnosis, treatments, follow up, signs of recurrence and management of late side effects Convenience sample Sample included participants up to 2 years post-treatment No control group Anxiety scores in sample were low using GAD-7
Ganz, 2003 [17]	To evaluate QOL and reproductive health outcomes in younger female breast cancer survivors	Cross sectional	577 women with stage 0, I, or II breast cancer who ranged in age from 30 to 61.6 years and were also disease-free survivors for 2 to 10 years	577 women	The Breast Cancer Prevention Trial Symptom Checklist, questions adapted from the Study of Women Across the Nation, RAND Short-form (SF)-36, Ladder of Life Scale, Center for Epidemiologic Studies–Depression Scale, and Sexual Activity Questionnaire	Medical and demographic factors, health-related QOL, mood, outlook on life, and reproductive health outcomes.	19 comorbid conditions that range from serious events such as stroke and heart attack, thyroid conditions, diabetes, high blood pressure, depression, and osteoarthritis. No specific questions on presence or absence of late-effects. The breast cancer prevention trial symptom checklist of 42 everyday problems (such as hot flashes, headaches, vaginal dryness, breast tenderness) was included.	Multiple regression analyses predicting QOL demonstrated better outcomes in African-American women, married, or partnered women, and women with better emotional and physical functioning, whereas women who reported greater vulnerability had poorer QOL.	Overall QOL in younger women who survive breast cancer is good, but there is evidence of increased emotional disruption, especially among the youngest women.	Both psychological and medical outcomes as well as comorbid conditions of cancer survivors were included. A diverse racial ethnic study sample (e.g., African Americans 11.6%; Hispanics 7.3%, and Asians 8.5%). No specific questions on late-effects.
Geller, 2014 [18]	To identify the needs and unmet needs of the growing number of adult cancer survivors.	Cross-sectional	Survey participants included 1668 individuals invited from the survivor registry; 65.7% were ages 60 or older and 61.9% were women.	1668 participants	Modified Cancer Survivors’ Unmet Needs (CaSun) instrument, which is an established measure of unmet needs for survivors.	53 specific needs in 5 domains: emotional, social, spiritual, economic and legal domains	Late effects after treatment was considered an unmet need	30.2% had at least one unmet need in the emotional, social, and spiritual I domain; just 14.4% had at least one unmet need in the economic and legal domain. The most commonly identified individual unmet needs were in the E and the information (I) domains and included “help reducing stress” (14.8% of all respondents) and “information about possible after effects of treatment” (14.4%).	Most needs of these longer-term survivors were met, but substantial proportions of survivors identified unmet needs. Unmet needs such as information about late and long-term adverse effects	Large sample with a comprehensive list of specific needs of cancer survivors. Needs that were unmet were just listed out with no details provided. There were no details regarding timing, frequency, duration, and other health problems.
Harrington, 2010 [19]	To assess late-effects and/or long-term psychosocial symptoms associated with cancer survivorship	Systematic review	69 studies based on four types of cancer- breast (n = 39), prostate (not reported), rectal/colon (not reported), gynecologic (n = 30)	69 reported studies			Cognitive limitations, depression/ anxiety, fatigue, pain/functional limitations, sexual function, sleep problems.	Regardless of the type of cancer and treatment, the most commonly reported symptoms include fatigue (over 50% for prostate cancer) (17–33% gynecological cancers) and depression/anxiety (14% -28% for gynecological cancers (breast cancer 30%)).	Fatigue and depression/anxiety were most reported late effects across the top four cancer diagnoses reported in the systematic studies	Considered late effects at various time points after treatment (immediately after treatment, 6, 6–12, and 1–2 years post treatment) Presented the top four most prevalent cancers. No details regarding timing, frequency, duration, and other health problems.
Lavoie Smith, 2012 [20]	To pilot test a Web-based cancer survivor needs assessment survey.	Cross-sectional	Cancer Survivors who completed survey within 5 months. They were predominantly white females.	547 participants	CS-WEBS	Survivor characteristics, physical and psychological needs, economic, social and spiritual needs	Comprehensive list of late effects	Participants reported fatigue (47%), forgetfulness (39%), joint pain (34%), anxiety (31%), trouble sleeping (28%), peripheral neuropathy (27%), inflexibility (23%), and weight gain (23%). Survivors with non-breast solid tumor malignancies reported more problems than those with breast or hematologic malignancies (P range = .037 to < .0001). Most survivors requested assistance for losing weight (74.2%), decreasing fatigue (50%), and improving flexibility (69.3%), sleep (68.5%), and memory (60.2%).	Survivors struggled with many enduring problems. Web based technology will assist with unmet needs.	Diverse population of cancer survivors with varied cancer diagnoses. They compared three different sampling approaches to reach a diverse sample. Tested three prior survey prototypes before using the final CS-WEBS online survey. Analyzed survivor needs by cancer type, survivorship phase, and years since treatment. A cross-sectional study design. Despite efforts to accrue a representative sample, this did not occur. The sample had 98% white females. High proportion of patients with breast cancer. Low response rate. Individuals with limited computer/internet experience were underrepresented. Self-reported cancer diagnoses stage and treatment information.
Ness, 2013 [21]	To evaluate the most prevalent physical, social, emotional, and spiritual concerns of cancer survivors.	Cross sectional	337 cancer survivors regardless of diagnosis or time since diagnosis were included; Predominantly Caucasians and female with median age of 63 years.	337 cancer survivors	Mayo clinic questionnaire	Physical, social, emotional, and spiritual, and other concerns and overall quality of life	Sexual issues, osteoporosis and bone health, memory and concentration, peripheral neuropathy, balance, lymphedema, fertility issues, fatigue, sleep disturbances, concerns about long term effects	Extreme concerns for cancer survivors were fear of recurrence (17%), fatigue and financial concerns (12%), long term effects about treatment, peripheral neuropathy and sexual issues (11%), finances, hot flashes, and osteoporosis/bone health (10%). Living with uncertainty (9%), and sleep disturbances (8%).	Fatigue and fear of recurrence were lasting concerns across the survivorship trajectory.	Several physical and psychosocial relevant concerns (late-effects were assessed). Adequate sample size Conducted this study to Identify areas where RNs can be proactive The sample was primarily female Caucasian. Survivors reported the concerns but not necessarily if they experienced them.
Rechis, 2010 [22]	To omprehend-sively assess the physical, emotional and practical needs of survivorship post-treatment. Further, the survey gathered information about why some post-treatment survivors did not receive care and, if they did receive care, who provided it.	Cross-sectional		The survey was created for patients diagnosed who completed cancer treatment or are still being managed with Tamoxifen. A total of 2,307 individuals were included in the survey analysis.	LIVESTRONG survey	1) physical concerns, 2) emotional concerns, 3) practical concerns, 4) positive experiences with cancer and 5) resources	No late effects presented	Physical Concerns 91 percent of the survey respondents (2,099) indicated that they had experienced one or more physical concerns since their cancer treatment was completed. The three most frequently selected collections were: 1) energy and rest, 2) concentration and 3) sexual functioning and satisfaction.	Over 90% of the sample experienced physical and/or mental concerns	For the survivors in this survey, in particular for emotional and practical concerns, many did not receive help for their post-treatment This survey contains questions on late effects. There is no information on timing, frequency, duration, other health problems, Nothing on minority and medically underserved populations The population was very narrow since the respondents were AYA cancer survivors, diagnosed between 15–39 years. Concerns.
								Mental concerns A total of 96 percent of the survey respondents (2,214) indicated that they had experienced one or more emotional concerns since their cancer treatment was completed. The three most frequently selected areas of emotional concerns were: 1) fear of recurrence of cancer, 2) grief and identity issues and 3) concerns about personal appearance.		
								Practical Concerns A total of 75 percent of these cancer survivors (1,719) indicated that they had experienced one or more practical concerns since their cancer treatment was completed.		
Rocque, 2014 [23]	To assess breast cancer survivors’ knowledge of cancer diagnosis, treatment, side effects, and long-term toxicities.	Randomi-zed control trial	38 patients (median age 57 years) diagnosed with stage 0-III breast cancer and completed active treatment. A total of 19 patients were in the intervention group and 19 in the control group.	38 baseline 16 intervention	Wisconsin Survey of Diagnosis and Management in Breast cancer (WiSDOM-B)	Characteristics of cancer at diagnosis, treatments rendered, long-term toxicities and side effects, and follow-up recommendations.	No late effects presented	Baseline knowledge was poor which increased following receipt of SCP (68.4vs. 74.4%), albeit not statistically significant.	WISDOM-B is a useful tool for assessing the impact of care plans on survivor knowledge.	Included side effects. Did not include prognosis, other ailments or side effects. Focused on knowledge of cancer survivors before and after administering SCP. Did not include prognosis, other ailments or side effects. Focused on knowledge of cancer survivors before and after administering SCP. Only included breast cancer patients <5 years from diagnosis. Did include side effects. Small sample size Did not specify if pre-or post-menopausal or if infertile.
Smith, 2006 [24]	To understand the quality of life (QOL) of cancer survivors	Longitudinal and Cross-sectional	61,847 cancer survivors Study of Cancer Survivor 1 (SCS 1): diagnosed with one of 10 common cancers (prostate, breast, lung, colorectal, bladder, non-Hodgkin lymphoma, skin melanoma, kidney, ovarian, and uterine) diagnosed during 12-month eligibility period Study of Cancer Survivor 2 (SCS 2): diagnosed with one of 6 cancers: prostate, female breast, colorectal, bladder, melanoma, and uterine either 2, 5, or 10 years prior to sampling.	Median response rate 34.9% completed questionnaires	Satisfaction with Life Domains Scale-Cancer, Cancer Problems in Living scale (psychological, physical, and concerns about community integration problems), Modified Rotterdam Symptom Checklist, Functional Assessment of Chronic Illness Therapy-Spiritual well-being (FACIT), Medical Outcomes Study, Multidimensional scale of perceived social support, and POMS 37	Symptom assessment, late medical event, Quality of Life, cancer recurrence, Comorbidities at study entry, lifestyle habits (smoking, alcohol use, diet, physical activity), and treatment (surgery, radiation, chemotherapy, hormonal therapy, bone marrow/stem cell transplant).	No late effects	Compared response rates by different demographics but there is no statistical analysis results provided	The two surveys provide a large demographically, diagnostically, geographically diverse database on cancer survivorship. Future reports will compare QOL survivors at different points in time.	Huge study sample Multiple types of cancer, multiple questionnaires Retrospective cohort. Omitted the entire epidemiology of late effects (onset, timing, frequency, duration, interaction of late effects, triggers and etiology.)

### Study quality assessment

Study quality assessment for each study was measured on a scale of zero to six (i.e., 0 = poor quality; 6 = highest quality. Only one study was rated with a very good quality assessment score of 5 [23]. Six studies were rated as good with a score of 4 [15, 18–22]. Four studies were rated as poor-quality assessment with a score of ≤2 [14, 16, 17, 24]. The details of the study design and study quality are presented in Table 2. The scoring system was devised by the authors. The table is organized by total quality score from lowest to highest.

**Table 2:** Results of quality assessment of included studies.

	Was the study question or objective clearly stated?	Were the subjects comparable within each study?	Were the outcome measures (late-effects) clearly defined, valid, reliable with adequate details (frequency, duration), and implemented consistently across all study participants?	Were details regarding the exposure (cancer survivorship) provided?	Was the questionnaire relevant to late effects?	Was the length of follow-up adequate?	Total score
Cancer Experience Registry, 2017 [14]	1	0	0	1	0	0	**2**
Curcio, 2012 [16]	1	0	0	1	0	0	**2**
Ganz, 2003 [17]	1	0	0	1	0	0	**2**
Smith, 2006 [24]	1	0	0	0	0	1	**2**
Childhood Cancer Survivor Study [15]	1	1	0	1	0	1	**4**
Geller, 2014 [18]	1	1	1	1	0	0	**4**
Harrington, 2010 [19]	1	0	1	0	1	1	**4**
Lavoie, 2012 [20]	1	0	1	1	1	0	**4**
Ness, 2013 [21]	1	0	1	1	1	0	**4**
Rechis, 2010 [22]	1	1	0	1	1	0	**4**
Rocque, 2014 [23]	1	1	0	1	1	1	**5**

0 = “No” and 1 = “Yes”

There were five cross sectional studies, two studies combining cross-sectional and longitudinal designs, one randomized clinical study, one longitudinal study, one retrospective cohort, and one systematic review. Sample sizes ranged from small (range 16–30), to mid-size, (range 337–577) to large (range 1,668–14,054).

All of the research questions and objectives were clearly stated. Several studies [15, 16, 19–22, 24] intentionally included multiple cancer diagnoses to appeal to a broader audience. Hence, it was difficult to compare the cancer survivors within and across studies. The remaining studies classified late effects as treatment effects, concerns, long-term effects, and unmet needs. The instruments that best captured late effects were created by Rocque [23], St. Jude’s [15], Rechis [22], Lavoie [20], Geller [18], and Ness [21]. Rocque’s study encompassed diagnosis, treatment, chronic side effects, and late side effects, but only among breast cancer survivors [23]. The major limitation of this study arose from “the dictionary of late or chronic side effects” developed by breast oncology specialists at the U of Wisconsin Carbone Cancer Center. Chronic side effects from surgical treatments (e.g., lumpectomy, mastectomy, sentinel lymph node biopsy) were presented [23]; however, there were no late effects revealed. Additionally, late effects related to radiation and endocrine therapy were not disclosed, apart from tamoxifen being linked to uterine cancer. However, late effects as a result of chemotherapeutic drugs were specified. For example, Epirubicin listed infertility or menopause, heart problems, and leukemia; carboplatin was grouped with infertility or menopause and neuropathy; and doxorubicin was put together with infertility or menopause, heart problems, and leukemia.

The St. Jude’s Childhood Cancer Survivor Study (CCSS) [15] is a component of the Long-Term Follow-Up Study. The CCSS is a retrospective cohort of 35,923 childhood cancer survivors diagnosed between 1970 and 1999 [25]. The current review determined that the St. Jude’s questionnaire was one of the higher quality questionnaires because it queried specific questions on late effects since May 1995 on medical conditions of the heart or lungs (e.g., blood clot), hearing/vision/nervous system, fatigue, sexual health, and cancer recurrences among pediatric cancer survivors. Additionally, there was one question cited in the breast cancer questionnaire, which pertained to the survivor developing a health problem several years later, which was related to their previous cancer treatment. Nevertheless, there were several organs omitted (e.g., gastrointestinal, genitourinary, central nervous system, musculoskeletal, thyroid, lymphatic, immune) from the CCSS questionnaire, the questions on late effects were cursory (i.e., present or absent), and the study sample only included childhood cancer survivors. Furthermore, emphasis was placed on long-term complications for one type of pediatric cancer (e.g., pediatric astrocytoma) [26]. The authors state that monitoring of late effects is important however, this was omitted from the results. Hence, the Childhood Cancer Survivor study did not provide a comprehensive list of questions pertaining to late effects of all organs; rather it only focused on four conditions within pediatric cancer survivors.

The LIVESTRONG survey by Rechis was created to better understand experiences after completion of cancer treatment [22]. It provided information on 1,719 survivors’ physical concerns including 1) Concentration, 2) Energy and rest, 3) Heart problems, 4) Infertility, 5) Lungs and breathing (short of breath), 6) Lymphedema, 7) Neuropathy, 8) Oral (tooth decay), 9) Pain, 10) Sexual functioning and satisfaction, 11) Thyroid function, 12) Urinary incontinence, and 13) Hearing loss. These concerns appeared to be synonymous with commonly occurring late effects, particularly because the participants had the opportunity to seek help for their complaints. The survey also ascertained emotional concerns including: 1. Faith and spirituality, 2. Fear of recurrence, 3. Personal appearance, 4. Personal and social relationships, 5. Sadness and depression. The final category for the LIVESTRONG survey consisted of Practical concerns including 1) Debt, 2) Insurance, 3) Employment issues, and 4) School issues. LIVESTRONG contained the premier questionnaire because it concentrated on numerous medical, psychosocial (emotional), and practical concerns. It also captured information regarding whether respondents received care for their complaints. However, the information collected on concerns was tabulated by participants selecting all pertinent options that apply (e.g., I have had trouble with my heart/lungs, I have been told by a doctor that I have heart problems, lung problems) with no additional details. Additionally, this study was cross-sectional.

The main objective of Lavoie’s study was to pilot test a Web-based cancer survivor (CS-WEBS) needs assessment survey [20]. The CS-WEBS survey was more focused on measuring a comprehensive list of needs of cancer treatment than other scored instruments. “Needs” appeared to be synonymous with symptoms that were present but are not necessarily bothersome. For example, “I have numbness and tingling in my hands and feet,” “I have this problem,” “I have this problem but it is not bothering me,” “I don’t have this problem,” and “I don’t have this problem because I am getting help for it.” The participants were predominantly white (98%) women (70%). One of the major limitations of this study was the lack of characterization of symptoms (e.g., tingling, anxious, depressed, tired, chest pain) of late effects.

Both Geller [18] and Ness [21] evaluated concerns rather than actual late effects experiences of cancer survivors. Ness’s cross-sectional survey [21] was used to assess the most prevalent physical, social, emotional, and spiritual concerns of cancer survivors. The sample consisted of survivors actively seeking cancer-related information and documented whether they were concerned about specific symptoms [21]. However, the study did not assess whether survivors actually experienced specific late effects.

Geller’s large community-based cancer survivor study, which used a modified version of the CaSun instrument [27], assessed 53 specific needs of cancer survivors [18]. This study team also determined whether the identified needs were met. The researchers did not assess which specific late effects the survivors were afflicted with at the time of the survey.

In contrast, Smith [24], and the Cancer Experience Registry Index study [14] did not include specific questions on late effects. Ganz [17] evaluated quality of life, nineteen comorbid conditions, and 42 everyday problems/symptoms; however, there were also no questions on late effects.

## Discussion

This systematic review summarizes the existence of all cancer survivorship instruments that assess the development, progression, and treatment of late effects among cancer survivors.

Existing instruments that best captured late effects were created by Rocque [23], St. Jude’s [15], Rechis [22], Lavoie [20], Geller [18], and Ness [21]. However, taken in totality, these six studies [15, 18, 20–23] did not adequately address the prevalence, etiology, characteristics (i.e., onset/timing, frequency, and duration), management, and prevention of late effects.

Although this systematic review used the best available evidence, there were several limitations. The exposure definitions and outcome measurements were defined differently across the individual studies. The studies all used different self-report instruments. There was no validation with medical records to confirm whether the survivor reported the late effect to their oncologist or primary care physician. The instruments were only distributed once, and thus, the majority of studies were cross-sectional, except for the CCSS study [15], as well as Curcio [16], and Rocque’s studies [23].

None of the instruments were customized by the type of cancer, age at treatment, and choice of treatment that would determine which specific late effects may be relevant. Furthermore, there was no information on timing, frequency, and duration of late effects in any of the questionnaires.

Other health problems of the cancer survivors were not taken into account, apart from CCSS [15] and LIVESTRONG [22] studies. Finally, there was no information on minority and medically underserved populations or non-English speaking cancer survivors.

One main overarching problem was the overlap of terms within all eleven papers, including treatment effects, late effects, long-term effects, unmet needs, and concerns (implying that the patient was worried but may not have experienced the late effect). Hence, it is evident that there is a lack of agreement about these important terms within the literature. A consensus should be developed so that each concept can be appropriately studied. Researchers should begin to tease out the differences between late effects and unmet needs. For example, the most frequently reported unmet needs of Australian cancer survivors were for help with psychosocial issues, including fear of cancer recurrence, uncertainty about the future, worry about partners, friends, and families, help to reduce stress, and sexual changes [28]. All of these factors relate to cancer survivors expressing a demand perceived by the patient that was not adequately met by the health care system [29]. A survivors’ opinion/impression or viewpoint is different from them actually experiencing physical or mental late effects. Hence, in the future, the presence of late effects and/or unmet needs should be presented separately, and categorized based on the cancer diagnosis, and time since completion of treatment, since both could vary considerably based on the initial years (1–2 years) compared to later years (5 or more years).

Finally, there is currently no comprehensive questionnaire that captures all of the relevant details of late effects (e.g., type, frequency, onset, duration) across the cancer survivorship continuum nor that tracks the evolution, chain of events, or interrelatedness of multiple late effects. Because these details are missing it is difficult to differentiate between late and long-term effects as well as identify, diagnose, manage and ultimately prevent late effects in cancer survivors.

## Supporting information

S1 Data Search strategy for PubMed. (DOCX)

S1 Fig PRISMA 2009 checklist. (DOC)
