# Supplementary material for: Existence of late-effects instruments for cancer survivors: A systematic review
Source: PLoS One. 2020 Feb 24;15(2):e0229222. doi: 10.1371/journal.pone.0229222 (PMC7039461; doi:10.1371/journal.pone.0229222)
Supplement: S1 Data — (DOCX) [file pone.0229222.s001.docx]

**Search strategy for PubMed**

“((late effects AND cancer survivor) AND (instrument OR questionnaire OR survey)))”

The search dates were set at default without any restrictions/filters.
